# Cancer

**Published:** 1906-02-17

**Authors:** 


### CANCER.

(*Continued from page* 322.)

**Cancer and the Thoracic Duct.**—After reference to certain reported cases in which abdominal cancer had spread to the thorax by way of the thoracic duct, Raw [11] records an instance of this condition. The history was that the patient, a man of forty-six, previously healthy, had for six months suffered from anæmia, obstinate constipation, and weakness. For two months he had observed a brawny inflamed swelling in the left supraclavicular fossa, with proptosis, pain and loss of sight in the left eye, and headache. No further symptoms were elicited in hospital, except some tenderness and resistance over the spleen, and a diagnosis of lymphadenoma was made. Post-mortem a primary growth was found in the head of the pancreas which pressed on the splenic flexure of the colon. The thoracic duct in the abdomen was not examined, but in the thorax its termination as well as the glands on the left side of the neck were found infiltrated with growth. There was also a growth behind the left eye. Raw states that the glands on the left side of the neck are more frequently involved in cancerous metastases than those on the right, and is inclined to attribute this to infection of the thoracic duct.

**Endothelioma.**—This term has been applied to certain malignant growths having their origin, as is supposed, from the endothelium of lymphatics and blood-vessels. The cells of which these growths are composed seem to hold a middle position between sarcoma and carcinoma, resembling sometimes one type and sometimes another. The difficulties which arise in properly classifying them are due to their doubtful histological position, and to the fact that in some cases a primary growth of epithelial origin may have been overlooked. Sturmdorf [12] has reported a case in which at an operation a mass was removed from the omentum, which appeared histologically to be an alveolar carcinoma. The peritoneum was studded with numerous nodules of varying size, and there was a serosanguineous effusion into its cavity. No evidence of any primary tumour could be found. The difficulty here was to account for the growth of a carcinomatous tumour from a structure possessing no elements of an epithelial type. Sturmdorf prefers to consider the growth a primary carcinoma of the omentum, owing to the doubtful histological position of endothelial tumours, and names the peritoneal condition a "peritonitis carcinomatosa," analogous to tuberculous peritonitis. Two cases recently reported by

Bushnell and Hinds[13] may usefully be compared with this. One, in the opinion of the authors, was undoubtedly a case of chronic inflammatory hyperplasia of all the coats of the stomach, but it suggests to them the possibility of gradations between chronic inflammation and true carcinoma. They quote various published records of transformation of one type of cell into another, and in view of the frequency of ulceration and metastases in primary sarcoma of the stomach, think that this case might closely resemble some examples of that condition. Their other case showed a very diffuse infiltration of the stomach and large and small intestines, and probably of the uterus and bladder. The condition resembled that described as "carcinomatosis," implying a diffuse outbreak of malignant growth from numerous foci at once. The authors note that the arrangement of the cells in relation to the lymphatics suggests that they may be termed endothelioma. But on the other hand, they quote cases in which so-called endothelial growths have subsequently been proved to be secondary to a scirrhus growth of the stomach. They thus appear to agree with Sturmdorf in discrediting "endotheliomata," but they apparently hold that multiple growths are really examples of Handley's "permeation by lymphatics" and not a true carcinomatosis of an inflammatory character. The three cases are interesting examples of the difficulties which surround the investigation of malignant disease.

**Hydrochloric Acid and Cancer.**—The absence of HCl in cases of gastric cancer is well recognised, but Moore, Alexander, and Kelly[14] have recently found evidence of its marked diminution or absence in cases of malignant disease in other organs. The theory of its suppression is considered by the authors to be inadequate. It has been held that in consequence of the dilatation and irritation of the stomach the normal secretion is suppressed and a catarrhal exudation occurs. But in other cases of dilatation not due to malignant growth HCl has been shown to be present, whereas it is absent quite early in cases of small malignant growths of the stomach when no ulceration or dilatation has occurred. In 17 cases of carcinoma of other organs they found the gastric HCl absent in about two-thirds, and much diminished in all the rest save one, in which it was present to the amount of one-fifth of the normal average. The total acidity was also invariably very low, and inorganic acids combined with organic bases was shown to be present. As far as could be determined the removal of the growth did not cause any increase in acid secretion. But the interest of these observations does not end here. The authors show that the "effective acidity" is in all cases greatly below normal. The "effective acidity" means the amount of hydrogen in a dissociated condition (hydrogen ions) present, capable of hydrolytic action. This being uniformly low, it is suggested that in carcinoma the "effective acidity," or in other words the concentration of hydrogen ions in the plasma is correspondingly low. Blood plasma contains both hydrogen and hydroxyl ions, and if the former are low the latter may be increased. There is at present no method capable of proving this. The authors, however, call attention to the well-known experiments of Loeb, who showed that slight increase in the alkalinity of the surrounding medium caused immense increase in the development and growth of the eggs of sea urchins. They thus surmise that slight increase in the concentration of hydroxyl ions in the plasma (effective alkalinity) may cause increased growth of a reversionary sexual type (heterotype mitosis) in certain cells predisposed thereto. In its relation to treatment this theory points to increase in the acidity of the plasma; but apart from the difficulty of such a process it would, as they remark, have to be pushed to the verge of acid intoxication.

[11] Brit. Med. Jour., June 24, 1905, p. 1380. [12] Amer. Jour. Med. Sci., April 1905, p. 633. [13] Brit. Med. Jour., Oct. 28, 1905, p. 1114. [14] Lancet, April 29, 1905, p. 1120.

